# Juvenile eosinophilic fasciitis: a single center case series

**DOI:** 10.1186/s12969-024-00960-w

**Published:** 2024-02-24

**Authors:** Leigh A. Stubbs, Oluwaseun Ogunbona, Emily Beil, Vibha Szafron, Adekunle Adesina, Sara Anvari, Jamie Lai, Andrea Ramirez, Matthew G. Ditzler, Marietta DeGuzman

**Affiliations:** 1 Department of Pediatrics, Division of Rheumatology, Baylor College of Medicine and Texas Children’s Hospital, Houston, TX USA; 2 Department of Pathology, Baylor College of Medicine and Texas Children’s Hospital, Houston, TX USA; 3 Department of Pathology and Genomic Medicine, Houston Methodist, Houston, TX USA; 4 Department of Pediatrics, Division of Rheumatology, Texas Children’s Hospital, Feigin Tower, 1102 Bates, Ste. 330, Houston, TX TX 77030 USA; 5 Department of Pediatrics, Division of Allergy and Immunology, Baylor College of Medicine and Texas Children’s Hospital, Houston, TX USA; 6 Department of Radiology, Baylor College of Medicine and Texas Children’s Hospital, Houston, TX USA

**Keywords:** Juvenile eosinophilic fasciitis, Juvenile localized scleroderma, Pediatric rheumatology, Autoimmune, Methotrexate

## Abstract

**Background:**

Eosinophilic fasciitis (EF) is a rare disease characterized by skin induration and musculoskeletal abnormalities. Diagnostic criteria for EF are based on adult populations. There is a need to expand the literature on EF in children due to limited reported cases and potential differences compared to adults.

**Methods:**

We conducted a retrospective review of medical records for six pediatric patients diagnosed with EF at our institution between November 2011 and April 2023. Inclusion criteria required patients to be under 18 years of age at the time of diagnosis and to have confirmed diagnosis through clinical history, imaging, and histology.

**Results:**

Most of our cohort were female (83%) and non-Hispanic white (50%). Age at diagnosis ranged from 4 to 16 years. Duration of symptoms before diagnosis varied from 1 to 12 months. Follow-up periods ranged from 14 to 123 months. Concurrent medical conditions included localized scleroderma, acquired thrombophilia, and juvenile idiopathic arthritis. Patients presented with progressive painful swelling, severe joint limitations, and positive prayer sign. Initial regimens involved corticosteroids and methotrexate. Hydroxychloroquine, immunoglobulin, mycophenolate mofetil, rituximab, and tocilizumab were also used depending on the patient’s disease severity and course.

**Conclusions:**

Juvenile EF may manifest as swelling and progressive induration without apparent skin abnormalities. Unlike adult populations, no underlying malignancies or associations with trauma were observed in our cohort. Our cases did not exhibit systemic involvement observed in previous studies on juvenile EF. While non-specific, the prayer sign may aid in early recognition of juvenile EF and help prevent long-term disability.

## Introduction

Eosinophilic fasciitis (EF) is a rare fibrosing disease *characterized* by acute painful swelling and progressive induration [1, 2]. Distal extremities are usually affected symmetrically or asymmetrically while the face and trunk are spared. Joint contractures, reduced mobility, and nerve compression lead to significant morbidity. Although the exact pathophysiology is still uncertain, immune-mediated mechanisms are involved [3, 4]. Various associations have been reported, such as intense exercise, trauma, radiotherapy, medications, autoimmune diseases, hematological disorders, infections, and malignancies. EF is often categorized within the spectrum of localized scleroderma (LS) and can be difficult to distinguish from other sclerosing skin disorders [2, 5, 6]. Delay in diagnosis can result in increased damage and reduced treatment response [7]. Misdiagnoses, such as systemic sclerosis, deep vein thrombosis (DVT), cellulitis, hypereosinophilic syndrome, pansclerotic morphea, stiff skin syndrome, or arthritis, can lead to invasive procedures or inappropriate treatments [7].

Diagnostic criteria for EF are not universally accepted or validated [8, 9]. The gold standard remains a full-thickness wedge biopsy demonstrating thickened fascia including lymphocytes and macrophages, with or without eosinophils. Supportive laboratory features include elevated eosinophil counts, sedimentation rate (ESR), hypergammaglobulinemia, and aldolase levels. Magnetic resonance imaging (MRI) can readily distinguish fascial thickening and is increasingly used for diagnosis and monitoring [10].

EF has been predominantly reported in middle-age white adults with limited pediatric cases [7, 11–15]. To expand upon and improve the understanding of the distinctive presentation of EF in the pediatric population, we report the, clinical characteristics, treatment, and course for six juvenile EF patients.

## Methods

We included patients under 18 years of age, diagnosed with confirmed clinical, MRI, and histopathological evidence of EF, from November 2011 to April 2023. *In addition to the Division of Rheumatology list, EPIC slicer dicer was utilized to identify these patients.* Data, including demographics, clinical characteristics, medications, pathology, laboratory results, and outcomes, were collected retrospectively from electronic medical records. Descriptive statistics were used to summarize the patient cohort. This study was approved by the Baylor College of Medicine Institutional Review Board.

The pathology slides for cases were reviewed. Hematoxylin and eosin (H&E) and immunohistochemical-stained sections were from formalin-fixed paraffin-embedded (FFPE) muscle biopsy samples with accompanying fascia. Immunohistochemical staining was performed following standard protocols including appropriate controls and validation for clinical immunohistochemistry tests.

## Results

### Demographics

There were six patients within our juvenile EF cohort (Table 1). The majority were female (83%). Patients identified as non-Hispanic white (50%), Hispanic white (33%), and non-Hispanic black (17%). The median age at initial presentation was 13 years (range 4–16 years). Patient 6 had a mild preceding COVID-19 infection. Otherwise, no specific triggers, such as direct injury, intense exercise, other infections, associated medications, or neoplasms, were identified.

**Table 1:** Juvenile eosinophilic fasciitis cohort patient characteristics. Bolded laboratory values are elevated above reference ranges

	**Patient 1**	**Patient 2**	**Patient 3**	**Patient 4**	**Patient 5**	**Patient 6**
**Sex**	M	F	F	F	F	F
**Race**	White	White	White	White	White	Black
**Ethnicity**	Non-Hispanic	Hispanic	Hispanic	Non-Hispanic	Non-Hispanic	Non-Hispanic
**Age at diagnosis *(years)***	14	4	10	12	16	16
**Current age *(years)***	28	14	15	15	18	18
**Symptom onset to diagnosis (months)**	12	12	6	12	< 1	1
**Follow up duration (months)**	58	123	52	28	23	14
**Other medical conditions** ***Prior to EF diagnosis*** At or after EF diagnosis	Localized scleroderma	Localized scleroderma, JIA	Localized scleroderma, JIA	22q11.23 deletion, Epilepsy JIA	Hashimoto thyroiditis, environmental and food allergies genetic factor XI deficiency	*None* Acquired hemophilia A
**Diagnosis labs** AEC (cells/uL, ref: 20–320) IgG (mg/dL, ref: 641–1353) *CK (U/L, ref:* > *295*) Aldolase (U/L, ref: 3.3 – 9.7) ESR (mm/hr, ref: < 1–20)	**700** **1990** *209* 7.1 14	**8206** **1888** *21* **12** **25**	**1390** **2100** *33* **11.4** **75**	**865** **2221** *20* **17.3** 6	**2130** 1330 28 **15.6** **21**	**390** **1720** 9 **34**
**Immunomodulation medications**	CS MTX HCQ	CS MTX HCQ IVIG TOCI	CS MTX HCQ IVIG RTX TOCI	CS MTX MMF	CS MTX HCQ IVIG	CS MTX RTX
**Current rheumatology medications**	None	TOCI	HCQ	MTX, MMF	HCQ, MTX	MTX
**History of recurrence of EF**	No	Yes	Yes	No	No	Yes

*Abbreviations*: *AEC* absolute eosinophil count, *CS* corticosteroids, *EF* eosinophilic fasciitis, *CK creatine kinase*, *ESR* erythrocyte sedimentation rate, *HCQ* hydroxychloroquine, *IgG* immunoglobulin G, *IVIG* intravenous immunoglobulin, *JIA* juvenile idiopathic arthritis, *MMF* mycophenolate mofetil, *MTX* methotrexate, *RTX* rituximab, *TOCI* tocilizumab

### Clinical, laboratory, and imaging findings

**Fig. 1:**
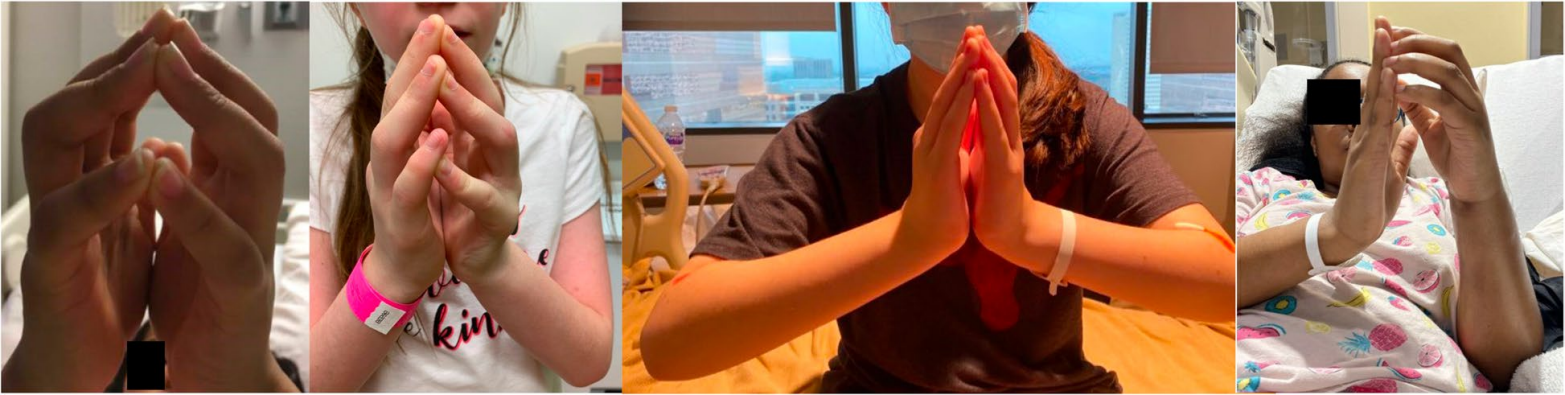
Positive prayer sign at initial presentation in juvenile eosinophilic fasciitis (EF). Here are examples of patient (**A**) 3, (**B**) 4, (**C**) 5, and (**D**) 6 demonstrating a positive prayer sign at initial diagnosis. In EF, a prayer sign is indicative of skin induration and fascial fibrosis resulting in joint contractures and tendon retraction

The median duration of symptoms before diagnosis was nine months (range 1–12 months). Most patients presented with bilateral, progressive painful swelling, induration, and thickening of the skin, leading to severe joint limitation. Patient 6 had unilateral involvement. All patients presented with a positive prayer sign (Fig. 1). The groove sign, a linear depression overlying veins with an elevated extremity, was not observed in any patients. Peau d’orange was noted *in* patient 1.

**Fig. 2:**
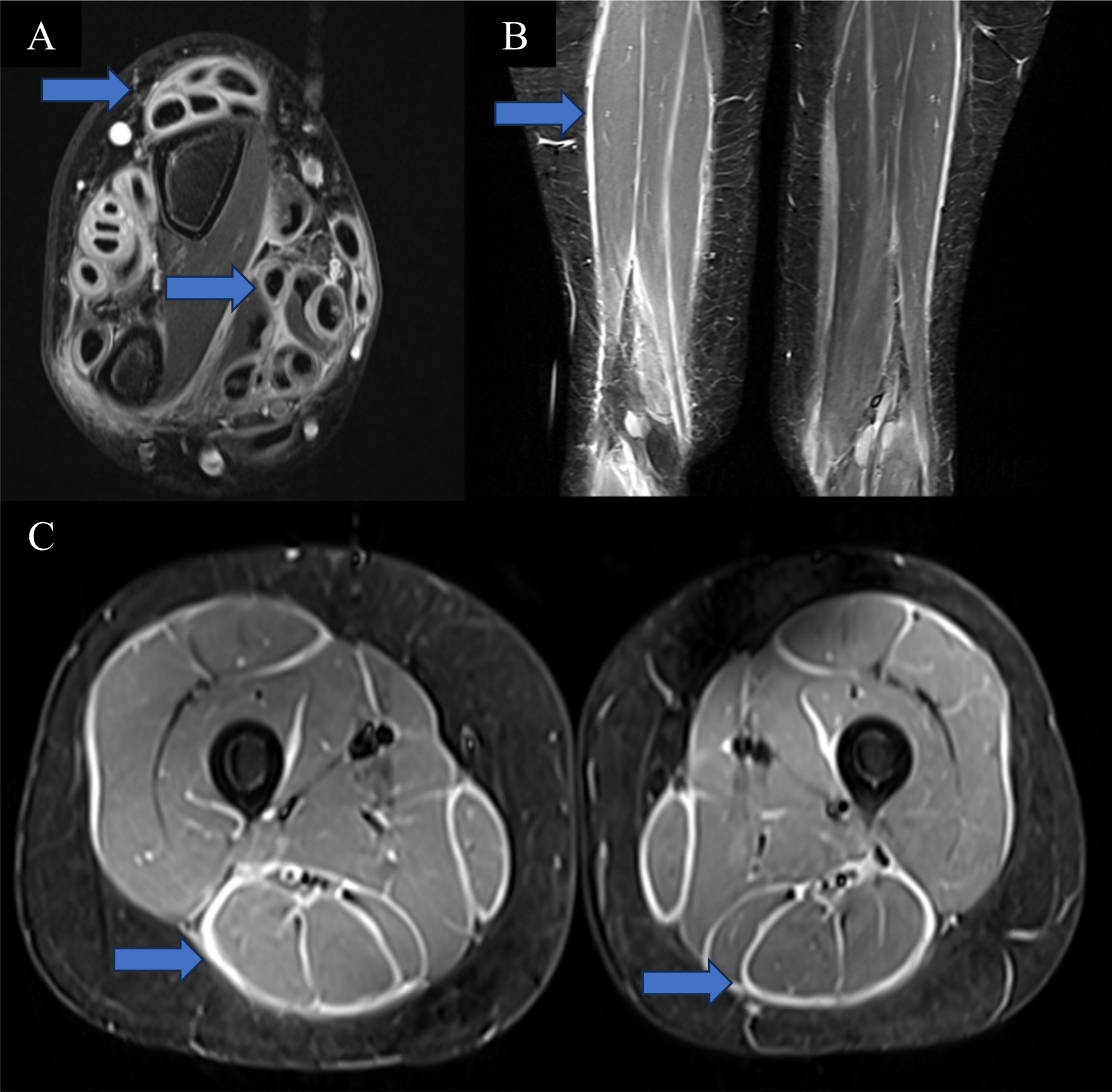
Examples of MRI findings. **A** Axial postcontrast MR image of the right wrist for patient 3 with marked tenosynovitis and myofasciitis. **B** Coronal and (**C**) axial STIR MR images of the bilateral thighs for patient 4 demonstrating extensive, symmetric fasciitis

Common laboratory features included elevated peripheral eosinophilia (100%), immunoglobulin G (IgG) (83%), aldolase (67%), and ESR (67%). All patients had an anti-nuclear antibody (ANA) panel available at diagnosis; four patients had a positive ANA (titers range: 1:80–1:1280) without any extractable nuclear antigen antibodies. Five out of the six patients had rheumatoid factor evaluated and tested negative. Patient 6 with acquired hemophilia A had normal prothrombin time, elevated partial thromboplastin time (82.3 s, reference: 23.1–36.3 s), decreased factor 8 level (< 1, reference: 50–150%), and presence of factor 8 inhibitor. All the patients had an MRI consistent with fasciitis (Fig. 2).

### Histopathologic features

**Fig. 3:**
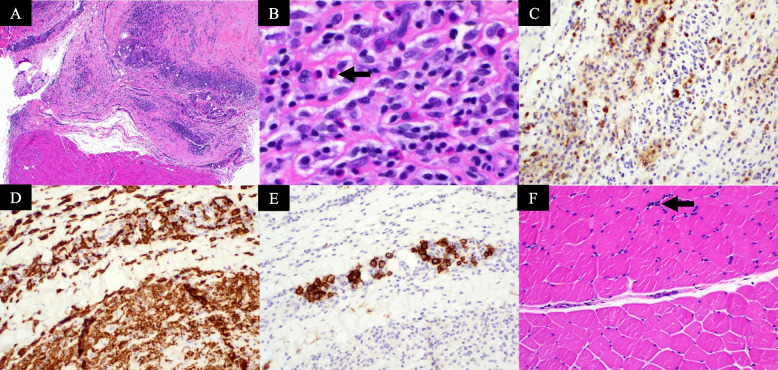
Representative eosinophilic fasciitis pathology characteristics. **A** Hematoxylin and eosin (H&E) section (patient 4, magnification, × 40) showing fascia with underlying muscle. The fascia shows thickening and moderate inflammatory cell infiltration. **B** H&E section (patient 3, magnification, × 400) showing extensive infiltration of fascia by inflammatory cells composed of lymphocytes, plasma cells, macrophages, and eosinophils. **C** CD45 (leukocyte common antigen, LCA) immunohistochemical stain (patient 4, magnification, × 200) highlighting the inflammatory cells. **D** CD163 immunohistochemical stain (patient 4, magnification, × 200) highlights macrophages. **E** CD138 immunohistochemical stain (patient 4, magnification, × 200), highlights plasma cells. **F** H&E section (patient 4, magnification, × 200) showing muscle with mild perimysial inflammation (black arrow)

H & E sections revealed inflammation that preferentially involved the fascia extending to the perimysium and *fascial* thickening (Fig. 3). In one case, there was focal destruction of myofibers. The intensity of inflammation ranged from mild to severe. Inflammatory infiltrates were composed of lymphocytes, plasma cells, and macrophages. Eosinophils were identified in four cases.

### Treatment and clinical outcomes

All patients received systemic corticosteroids and methotrexate. Other immunomodulatory medications were administered depending on the patient’s course, including hydroxychloroquine (*n* = 4), intravenous immunoglobulin (IVIG) (*n* = 3), tocilizumab (*n* = 2), rituximab (*n* = 2), and mycophenolate mofetil (*n* = 1).

Patient 1 was diagnosed at age 14 years and *whose treatment included a course of IV steroids, methotrexate and hydroxychloroquine. His treatment was interrupted by periods of nonadherence, and approximately two years after his EF diagnosis,* he had new generalized LS lesions. Oral methotrexate 25 mg weekly and hydroxychloroquine 400 mg daily *were continued*. Given the stability of his condition, he was transitioned to adult care and medications were discontinued. *Throughout his pediatric rheumatology care, there was* no recurrence of EF.

**Fig. 4:**
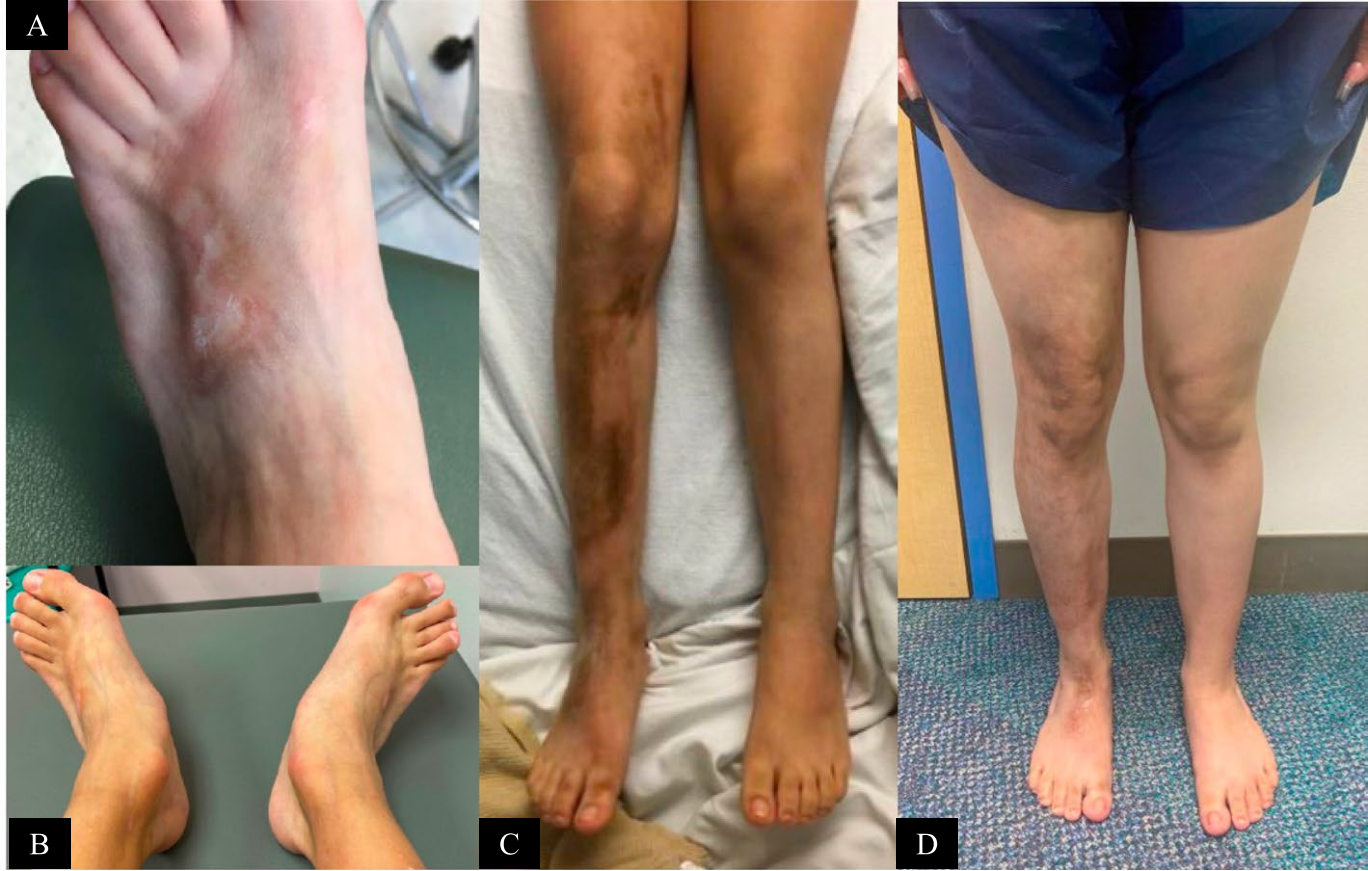
Features of localized scleroderma. **A** Patient 2’s damage features of localized scleroderma include visible venous pattern resulting from epidermal and dermal atrophy. There is a plaque of bound down, sclerotic skin causing hyperpigmentation, sclerotic bands, atrophy, and contractures. **B** As a sequela of fascial and tendon fibrosis as well as joint ankylosis, she also has bilateral severe flatfoot deformity with hindfoot valgus. **C** Patient 3’s initial diagnosis image including active right leg linear scleroderma with hyperpigmented plaques with central thickening surrounded by outer erythema. **D** Patient 3’s current physical examination shows late-stage morphea damage features including right lower extremity dermal atrophy with visible vessels, subcutaneous atrophy, dyspigmentation, decreased leg circumference, mild right ankle flexion contracture, and non-significant leg length difference (8 mm)

Patient 2 was diagnosed at the age of four *years* with a *morphea* and relapsing and remitting course of EF requiring intermittent corticosteroids *(IV methylprednisolone)*, methotrexate (15 mg/m^2^/weekly subcutaneous (SQ) injections), hydroxychloroquine (3 mg/kg/day), and IVIG (2 g/kg monthly). She had remission of *both morphea and* EF leading to a gradual taper of medications. At age 13, she developed JIA, involving bilateral shoulder and temporomandibular joint*s*; *MRI without and with contrast showed chronic inflammatory arthritis, with* erosive *changes*. After starting tocilizumab 162 mg SQ injections every 28 days, she achieved clinical remission. Notably, she has significant damage features of LS and EF (Fig. 4 A-B).

Patient 3 was diagnosed at age 10 years with extensive bilateral wrist/hand tenosynovitis, myofasciitis, and bilateral lower extremity fasciitis (Fig. 2 A). She was concurrently diagnosed with active linear LS of her right lower extremity (Fig. 4 C). She was treated with hydroxychloroquine (3 mg/kg/day), methylprednisolone (30 mg/kg IV for three days, then weekly for 12 weeks), IVIG (2 g/kg monthly), and methotrexate (15 mg/m^2^/weekly SQ injections). Given only a partial response, rituximab (750 mg/m^2^/dose two weeks apart for two doses) was added. Despite initial clinical improvement, her LS and EF relapsed, leading to the addition of tocilizumab (162 mg/dose IV every two weeks). For the past three years, she has maintained remission as confirmed by clinical examination and MRI. Subsequently, she has tapered off prednisone, methotrexate, and tocilizumab. Currently, she is on hydroxychloroquine with only LS damage features present (Fig. 4 D).

Patient 4 had a history of 22q11.23 deletion and epilepsy. EF was diagnosed at 12 years* of age* with extensive symmetric fasciitis (Fig. 2 B-C). She had limited and painful range of motion of her shoulders, elbows, wrists, fingers, hips, knees, and ankles. She was treated with prednisone (maximum dose of 40 mg daily, 1 mg/kg/day), IV methylprednisolone (30 mg/kg pulses weekly for four doses), and methotrexate (15 mg/m^2^/weekly SQ injections). This regimen led to a significant improvement in her joint limitation. While weaning corticosteroids, she developed skin changes and mild elevation of aldolase and lactate dehydrogenase, so mycophenolate mofetil (600 mg/m^2^/dose twice daily) was added leading to normalization of her labs, clinical features, and MRI. She tolerated a prednisone wean without issues. She remains in remission and is undergoing a gradual tapering of methotrexate.

Patient 5 had a history of Hashimoto thyroiditis, environmental allergies requiring immunotherapy, food allergies, and inherited mild factor XI deficiency. EF was diagnosed within three weeks of symptoms, including generalized edema, limitation of her bilateral shoulders, fingers, wrists, and decreased ability to squat. MRI of her bilateral thighs exhibited circumferential, uniform thickness edema-like signal along the intermuscular fascial planes diffusely about the pelvis and thighs. She was treated with IV methylprednisolone (1000 mg total for one dose), prednisone (maximum dose of 40 mg daily, approximately 1 mg/kg/day), IVIG (2 g/kg monthly for six doses), methotrexate (15 mg/m^2^/weekly oral), and hydroxychloroquine 200 mg daily (4 mg/kg/day). Within a month of treatment, her clinical symptoms and laboratory markers normalized. After six months of treatment, a repeat MRI showed interval resolution. She remains in remission on hydroxychloroquine and a slow wean of methotrexate.

Patient 6 was diagnosed with unilateral EF involving her left forearm, wrist, fingers, and lower extremity. Initially, she presented with left calf pain and swelling with negative DVT evaluation. She was misdiagnosed with a soft tissue infection. Her symptoms progressed over a month to include circumferential swelling of her left wrist, forearm, fingers, and lower extremity with associated limited range of motion. She also had scattered ecchymoses. MRI of her left upper and lower extremity demonstrated diffuse myositis and fasciitis. Given her diagnoses of EF and acquired hemophilia A, she was started on corticosteroids, rituximab, and methotrexate (25 mg weekly SQ injections). She was lost to specialty follow up care and received a truncated course of IV methylprednisolone (1000 mg weekly dose for two doses), prednisone (maximum: 40 mg daily), and rituximab (800 mg and then 1000 mg two weeks later) with improvement of her musculoskeletal symptoms and improvement of her factor 8 level. Fourteen months later, she presented with four days of acute onset bilateral upper and lower extremity swelling and was found to have peripheral eosinophilia and elevated CK, consistent with a flare of EF. She had no abnormal bruising and normal coagulation markers. She was given a three-day course of 1000 mg IV methylprednisolone, followed by prednisone taper, and started on methotrexate 25 mg SQ injections with notable improvement of her symptoms.

## Discussion

Our cohort of six patients aligns and expands upon existing juvenile EF literature. This cohort represents the largest juvenile EF cohort since Zulian and colleagues reported 10 cases within the largest juvenile LS cohort of 750 patients [6]. Otherwise, there have been smaller case series and case reports [12, 13, 15]. Similar to previous juvenile cohorts, there was a female predominance of patients. In comparison to the prior median onset age of eight (*range 1–17 years*), the median age within our cohort was older at 13 (range 4–16 years) [13]. Although there are reports of visceral involvement, such as mesenteric lymphadenopathy, hepatomegaly, splenomegaly, or pericardial effusion, this was not evident within our cohort. The characteristic histopathologic finding was the presence of variable degree of inflammation involving the fascia with little to no involvement of the underlying muscle. Of note, eosinophils were not always present in the observed inflammatory infiltrates.

Despite similarities in laboratory abnormalities, MRI findings, and histopathology, there are some distinctive differences between adult and juvenile EF. There have been numerous reported triggers and factors associated with adult EF. A history of intense physical exertion or trauma was found in 28–46% of adult patients [7, 8]. Within the juvenile EF literature and our cohort, this does not seem to be a significant trigger [13]. Moreover, common drug triggers, such as statins or ramipril, are less frequently used in the pediatric population. Adult EF has also been associated with solid neoplasms, hematologic disorders, autoimmune conditions, radiotherapy, burns, and infections [8]. Similar to our patient with preceding COVID-19, there are more reported pediatric cases with preceding non-specific infections [2]. Although one of our patients had acquired hemophilia, this is not one of the previously reported hematological associations. Notably, autoimmune diseases seem to be associated in both adult and juvenile EF patients. Specifically, concurrent morphea has been reported in 30–50% of adult EF patients and occurred in 50% of our pediatric cohort [7, 16, 17]. *EF had been described along the spectrum of scleroderma-like disease, its association with LS remains to be elucidated. With the currently described phenotypes of LS, EF perhaps belong to the more severe end of the morphea spectrum. Laboratory tests and skin biopsy are not necessary in majority of LS, while a histopathologic studies of full-thickness skin biopsy, with fascia and muscle tissues are required for the diagnosis of EF.*

In contrast to adult EF patients, juvenile EF patients less often present with the cutaneous manifestations such as the groove sign or peau d’orange whereas articular manifestations such as joint contractures, tendon retractions, and prayer sign are always reported [8, 13, 15]. Similar to juvenile LS, juvenile EF patients have increased reports of relapse/resistance to treatments and disabling outcomes compared to adult cohorts [13, 18].

There is no current standardized therapy for EF. While corticosteroids are considered first-line treatment, challenges may arise with prolonged use, partial response, or tapering. Adult studies have shown a higher rate of complete response with combination therapy of corticosteroids and methotrexate [7, 16, 17, 19]. Other steroid-sparing agents utilized include anti-tumor necrosis factor agents, azathioprine, cyclosporine, imatinib, IVIG, hydroxychloroquine, mycophenolate mofetil, rituximab, sirolimus, and tocilizumab in retrospective cases or case series [10, 12, 15, 17, 20–24]. Given its rarity, treatment from juvenile EF is adapted from treatment for juvenile LS and dermatomyositis [25–27]. Poor outcomes are associated with a diagnostic and treatment delay of greater than six months [7, 17]. The duration of therapy required is unclear. Relapses have responded well to resuming methotrexate [7]. Adjunctive early physical therapy helps to limit contractures and improve mobility [9, 10].

In conclusion, this study provides further insights into EF in the pediatric population. Despite limitations such as a small sample size and retrospective design, the findings contribute to a better understanding of the similarities and differences between adult and juvenile EF. This cohort highlights the importance of recognition of the musculoskeletal features which can lead to early diagnosis and appropriate treatment to prevent long-term damage and disability. While corticosteroids remain the first-line therapy, combination regimens including methotrexate and other immunomodulatory agents have shown promising outcomes. Further research is needed to establish standardized diagnostic criteria and optimal treatment approaches for EF in both adult and pediatric patients.

## Data Availability

The data supporting this study’s findings are available on request from the corresponding author. The data are not publicly available due to privacy and ethical restrictions.

## Abbreviations

AEC: Absolute eosinophil count
ANA: Anti-nuclear antibody
CS: Corticosteroids
DVT: Deep vein thrombosis
EF: Eosinophilic fasciitis
ESR: Erythrocyte sedimentation rate
FFPE: Formalin-fixed paraffin-embedded
HCQ: Hydroxychloroquine
H&E: Hematoxylin and eosin
IgG: Immunoglobulin G
IVIG: Intravenous immunoglobulin
JIA: Juvenile idiopathic arthritis
LS: Localized scleroderma
MMF: Mycophenolate mofetil
MRI: Magnetic resonance imaging
MTX: Methotrexate
RTX: Rituximab
TOCI: Tocilizumab
